# COD capture: a feasible option towards energy self-sufficient domestic wastewater treatment

**DOI:** 10.1038/srep25054

**Published:** 2016-04-28

**Authors:** Junfeng Wan, Jun Gu, Qian Zhao, Yu Liu

**Affiliations:** 1 School of Civil and Environmental Engineering, Nanyang Technological University, 50 Nanyang Avenue, 639798, Singapore; 2 Advanced Environmental Biotechnology Centre, Nanyang Environment and Water Research Institute, Nanyang Technological University, 1 Cleantech Loop, CleanTech One, 637141, Singapore; 3 School of Chemical Engineering and Energy, Zhengzhou University, 100 Science Avenue, 450001, P. R. China

## Abstract

Although the activated sludge process, one of the most remarkable engineering inventions in the 20^th^ century, has made significant contribution to wastewater reclamation in the past 100 years, its high energy consumption is posing a serious impact and challenge on the current wastewater industry worldwide and is also inevitably linked to the issue of global climate change. In this study, we argued that substantial improvement in the energy efficiency might be no longer achievable through further optimization of the activated sludge process. Instead, we should devote more effort to the development or the adoption of novel treatment configurations and emerging technologies. Of which an example is A-B process which can significantly improve the energy recovery potential at A-stage, while markedly reduces energy consumption at B-stage. Various configurations of A-B process with energy analysis are thus discussed. It appears highly possible to achieve an overall energy gain in WWTPs with A-B process as a core.

The conventional activated sludge (CAS) process has been employed for domestic wastewater treatment for more than 100 years, where organic matters are mainly converted to biomass and carbon dioxide. In practice, the CAS process has been extended and modified to handle soluble nutrients (e.g. nitrogen and phosphorus) present in domestic wastewater. One of such examples is the CAS-based various biological nitrogen removal (BNR) processes including the latest short-cut nitrification coupled with anaerobic ammonium oxidation (anammox) process^1^,^2^. These new development may lead to more sustainable and economically viable biological processes for nutrient removal. However, it should also be noted that the CAS process is receiving increasing pressure due to its low energy efficiency. For example, 35% of the energy efficiency was achieved in the Jurong Water Reclamation Plant of Singapore, whereas only 31% in the Gaobeidian Wastewater Treatment Plants of China^3^. It had been reported that about 3% of the annual electrical energy was consumed for wastewater treatment in USA^4^. Moreover, increasing in-plant energy consumption would be inevitable since discharge standards of effluent are being tightened in more and more countries due to the serious concerns on public health and environmental protection. These imply that energy-intensive treatment processes may possibly be required for meeting such needs. With no doubt, the substantial energy consumption in current wastewater treatment plants (WWTPs) is a critical issue that challenges the global wastewater treatment practice, especially when environmental sustainability is concerned.

In WWTPs, a substantial amount of electrical energy that generated essentially from fossil fuels is consumed to drive wastewater treatment. This is inevitably linked to the issue of global climate change. In China, about 114 million metric tons of carbon dioxide is generated and released annually from the production of electrical energy required to drive the WWTPs operation countrywide^5^. Meanwhile, a great amount of greenhouse gases, such as carbon dioxide, methane and nitrogen oxide, is produced during various biological conversions of organic and nitrogenous matters present in domestic wastewater. Obviously, substantial in-plant energy consumption will have considerable impact on the future wastewater industry in consideration of global climate change.

In practice, the chemical oxygen demand (COD) has been commonly employed to determine the total amount of organic matters in domestic wastewater. In the WWTPs with the CAS as core treatment technology, given a growth yield of 0.3–0.5 g dry biomass produced per gram COD removed, 1 kg COD removed will generate 0.3–0.5 kg dry biomass. As a result, a large amount of waste sludge is currently generated during biological treatment of domestic wastewater globally. In order to reduce electric energy input to a WWTP, the present practice is to recover energy from waste sludge through anaerobic digestion. Despite advanced development of anaerobic digestion technology, only about 30–50% of the total COD or volatile solids can be degraded towards biogas production, implying that the overall energy efficiency of anaerobic digestion process is very low due to the fact that microorganisms cannot directly utilize waste sludge, which instead should be hydrolyzed to soluble COD prior to anaerobic digestion. This indeed is the major drawback of the present WWTP settings in which soluble COD is first converted to biomass from which energy is subsequently recovered through anaerobic digestion at low energy efficiency. For example, the overall in-plant energy efficiency of 20–50% has been reported in various countries^3^,^6^,^7^,^8^. Therefore, there is an urgent global need to substantially improve the overall energy efficiency of WWTP by adopting innovative process configuration. Ideally, organic maters present in domestic wastewater should be captured as much as possible for direct anaerobic digestion prior to biological conversion to sludge. This in turn may lead to the paradigm shift of the current WWTP with the benefits of (i) enhanced energy recovery; (ii) reduced energy consumption associated with aeration and (iii) lesser excessive sludge production. In this study, we described that WWTP should be designed according to the concept of A-B process for significantly improving the energy recovery potential, while reducing in-plant energy consumption. Hence, various possible configurations of A-B process with energy analysis are discussed. With the advances in environmental microbiology along with recent development in autotrophic ammonia removal, A-B process appears to be a feasible option towards energy self-sufficiency in domestic wastewater treatment plant.

## Results

### Energy situation in current WWTPs

**Figure 1:**
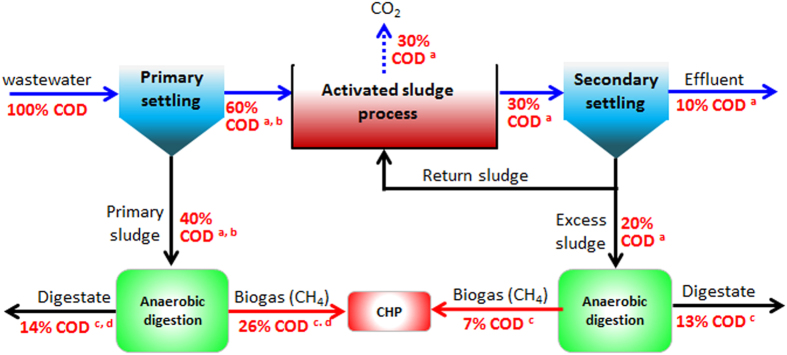
COD mass flow through a conventional domestic wastewater treatment plant. (**a**) data from Metcalf and Eddy^12^; (**b**) data from Rossle and Pretorius^18^; (**c**) data from Parkin and Owen^47^; (**d**) data from Miron *et al.*^48^.

Nowadays, domestic wastewater cannot be simply considered as “waste”, but a valuable resource full of energy, valuable materials and clean water. The current energy consumption in the conventional CAS process is normally in the range of 0.3 to 0.6 kWh per m^3^ of wastewater treated with an average of 0.45 kWh/m^3^ equivalent to 1620 kJ/m^3^,^3^,^9^ whereas a typicalCOD concentration in domestic wastewater is often around 500 mg/L. Therefore, the energy consumption in the conventional CAS process can be estimated as 1620 kJ m^−3^/500 g COD m^−3^ = 3.20 kJ/g COD. Theoretically, the potential energy in typical domestic wastewater has been estimated in the range of 14.7–17.8 kJ free energy/g COD, averaged at 16.2 kJ/g COD^10^,^11^ which is nearly 5-fold of electrical energy (i.e. 3.2 kJ/g COD) utilized to drive wastewater treatment in the CAS process. These numbers potentially suggest that WWTPs may be energy self-sufficient so long as 20% of the total energy in domestic wastewater could be completely converted to the electrical energy. Figure 1 illustrates the COD mass flow in a typical domestic wastewater treatment plant with the CAS as a core treatment unit. It is obvious that in such a process, the total energy that can potentially be recovered essentially comes from anaerobic digestions of both primary and secondary sludges.

As can be seen in Fig. 1, 26% and 7% of total COD from primary and secondary sludges could be converted to methane through anaerobic digestion, respectively. As theoretical chemical energy derived from one gram of methane-COD is about 13.9 kJ^11^, the total recoverable chemical energy may be calculated as the sum of (13.9 × 0.26) from primary sludge and (13.9 × 0.07) from secondary sludge, i.e. 4.58 kJ/g COD. However, it should be noted that only about 35% of the chemical energy of methane can be converted to electricity through its combustion^9^. Therefore, the maximum recoverable electrical energy from domestic wastewater through anaerobic digestion in WWTPs is about (4.58 × 0.35) = 1.60 kJ/g COD, which is the half of the electrical energy required for removing one gram COD (i.e. 3.20 kJ/g COD). These suggest that the maximum electrical energy recovered from wastewater can only offset at most 50% of total energy needed for the current WWTP operation (Fig. 1), which indeed is consistent with the previous estimate^9^. Zhou *et al.*^3^ conducted a series of comprehensive survey on the global energy situation in WWTPs, according to which only about 35% of energy efficiency was realized in the Jurong Water Reclamation Plant of Singapore, whereas only 31% in the Gaobeidian WWTP of China. As illustrated in Fig. 1, anaerobic digestion of primary sludge may contribute to about 78% of the total potential recoverable energy in WWTPs, although only 40% of total COD in domestic wastewater is captured in the primary settling tank. This in turn points to a fact that capture of COD prior to biological oxidation is essential for further improving the plant energy balance. Furthermore, the combined heat and power generation may be considered as an economically viable option for further enhancing the overall conversion efficiency of biogas to recoverable energy in terms of both electricity and heat. In fact, it also appears difficult to achieve energy-neutral or even more challenging for energy-positive operation if the CAS process continues to serve as the core unit of WWTPs. Given such a situation, the current debate on the situation forward is naturally towards the technological options that may lead to the paradigm shift of the WWTP’s operation from energy-negative to neutral and ultimately positive.

### A-B process for domestic wastewater treatment

#### Rational of A-B process

**Figure 2:**
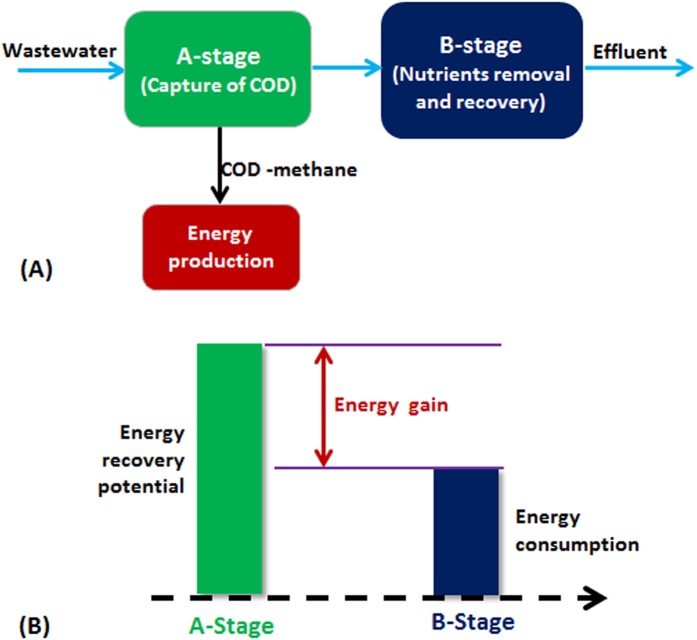
A general configuration of A-B process.

The central point towards energy recovery is to capture COD from wastewater as much as possible prior to its biological oxidation. As such, the present CAS process should be retrofitted into a two-stage system known as A-B process which is schematically elucidated in Fig. 2A. In A-B process, A-stage is specifically designed to maximize the capture of organic matters from domestic wastewater for direct anaerobic digestion prior to biological oxidation, whereas B-stage is mainly dedicated to handling nutrients. Theoretically, the electrical energy generated should be sufficient to drive the conventional WWTP operation (i.e. 3.2 kJ is required for removing one gram COD) if 65% of total COD in domestic wastewater could be captured in A-stage and converted to methane through anaerobic digestion, i.e. the electrical energy produced from one gram COD = 13.9 × 65% × 35% = 3.2 kJ. As a result of COD capture in A-stage, substantially reduced energy consumption would be expected in B-stage (Fig. 2A). The essence of A-B process is to improve energy recovery through COD capture prior to biological oxidation in A-stage, whereas to reduce energy consumption in B-stage (Fig. 2B). This is the only way possible to realize the energy self-sufficient operation of a WWTP.

**Figure 3:**
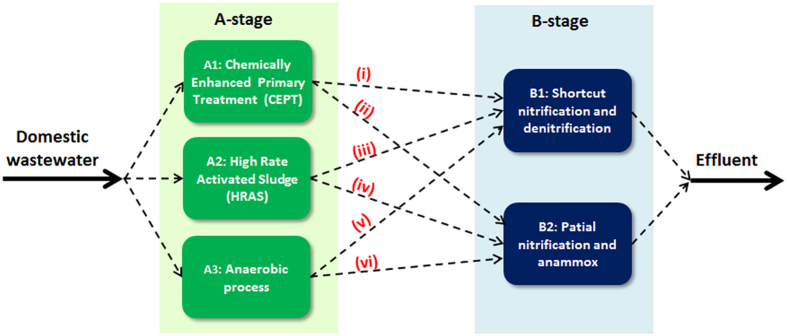
Possible combinations for A-B processes.

The conceptual A-B process (Fig. 2A) is specifically put forward for maximizing the recovery of the chemical energy from domestic wastewater, instead of transforming it to carbon dioxide during biological oxidation of COD with consummation of substantial amount of electrical energy. However, the feasible options of A-B process towards the process energy self-sufficiency are determined by the choices of A and B stages. In this regard, only three processes with various potentials of the capture of COD from wastewater may serve as A-stage, i.e. (i) chemically enhanced primary treatment (CEPT) process, (ii) high rate activated sludge (HRAS) process and (iii) anaerobic process. It appears from the literature that these three processes would be able to capture at least 60% of total COD in domestic wastewater. Obviously, if they are chosen to serve as A-stage of A-B process, much lesser COD would enter B-stage. According to the denitrification stoichiometry, about 2.86 gram COD should be required for completely denitrifying one gram NO_3_^−^-N^12^. These seem to suggest that the conventional nitrification and denitrification process could not be considered as a choice of B-stage due to the insufficient supply of soluble COD after the COD capture at A-stage. In fact, it appears from the literature that only two processes, i.e. shortcut nitrification-denitrification and partial nitrification combined with anammox processes, may meet the requirements of B-stage. Therefore, Fig. 3 further illustrates various feasible combinations of A-B process.

#### A1-B1 or A1-B2 process

CEPT process has been widely employed to treat domestic wastewater for decades. Up to 80% of TSS and 60% of total COD can be removed through CEPT in full-scale WWTPs^13^,^14^,^15^,^16^. Compared to the conventional primary settling, the quantity of sludge produced by CEPT was found to be increased by nearly 45%, of which 33% was due to enhanced solids capture^13^. Moreover, organic-rich CEPT sludge is more suitable for biogas production through anaerobic digestion^17^. However, it should be noted that CEPT is very poor in removing soluble COD from domestic wastewater, i.e. nearly all soluble COD which is at least 30% of total COD in domestic wastewater may enter into B-stage^18^. In case where CEPT serves as A-stage, soluble COD going into B-stage should be high enough for inhibiting the growth of anammox bacteria against heterotrophic denitrifying bacteria^19^,^20^. Therefore, it should be more reasonable to consider the shortcut nitrification-denitrification process as B-stage.

**Figure 4:**
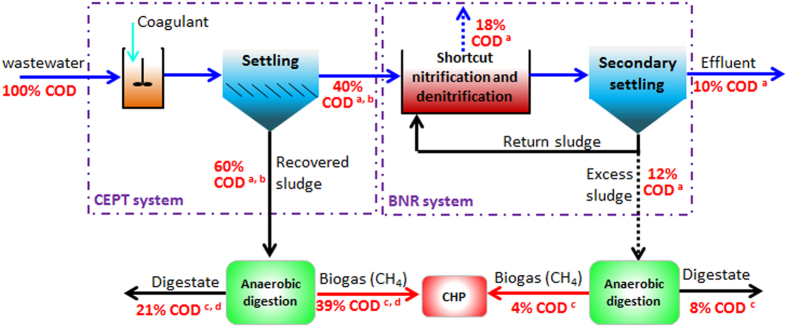
COD flow in A-B process with CEPT as A-stage and shortcut nitrification and denitrification as B-stage; (**a**) data from Metcalf and Eddy^12^; (**b**) data from Morrissey and Harleman^13^; (**c**) data from Parkin and Owen^47^; (**d**) data from Miron *et al.*^48^.

In the above discussion, the CEPT-shortcut nitrification-denitrification has been identified as a feasible configuration of A-B process. The COD mass flows in this process configuration are presented in Fig. 4. It appears that 43% of influent COD can be ultimately converted to biomethane, thus the maximum electrical energy potentially recovered from one gram of wastewater COD is calculated as (13.9 × 43% × 0.35) = 2.09 kJ/g COD. It had been reported that addition of chemical coagulant would not adversely affect the subsequent anaerobic digestion efficiency of wasted sludge^21^. It should also be noted that addition of coagulants to CEPT inevitably leads to an increased overall operation cost. According to Diamantis *et al.*^17^, the additional operational cost associated with coagulant was about 0.1 €/m^3^ (equivalent to 4 × 10^−4^ €/g COD) when the CEPT process was adapted for the pre-concentration of municipal wastewater. Thus, a detailed cost-benefit analysis should be conducted if CEPT was considered as A-stage in A-B process illustrated in Fig. 4.

#### A2-B1 or A2-B2 process

HRAS as a variant of the CAS process has attracted great attention due to its high capacity of COD capture at short sludge and hydraulic retention times, e.g. about 55–65% of organic matter could be retained in HRAS process at a SRT of 0.5 day^22^, wheras nitrification in HRAS should not be significant due to the shorter sludge retention time less than 3 days. As such, for a domestic wastewater with a typical COD concnetration of 500 mg/L, a substantial amount of COD at the concnetration level of 175 to 225 mg/L may eventually enter into B-stage, leading to a COD/N ratio of 7 to 9. In general, heterotrophic denitrifiers can easily outcompete anammox bacteria at the soluble COD/N ratio greater than 2^19^,^23^. Theroretically, 1.71 gram COD is required to denitrify 1 gram NO_2_^−^-N, i.e. COD remained after A-stage is sufficient for removing ammonia through shortcut nitrification-denitrification. It should also be pointed out that the success of the integrated HRAS- anammox process in the Strass WWTP, to a large extent, is due to the selectively retention and return of anammox bacteria harvested from the sidestream deammonification unit by cyclone or screens at B-stage^24^. In addition, the partial nitritation-anammox process could be employed as B-satge if the COD/NH_4_^+^ -N ratio was lowered to 1.9 in the HRAS process as A-stage^25^. Consequnetly, these seem to suggest that the A2-B2 process is more challenging than the A2-B1 process from the point of view of the system feasibility and stability.

**Figure 5:**
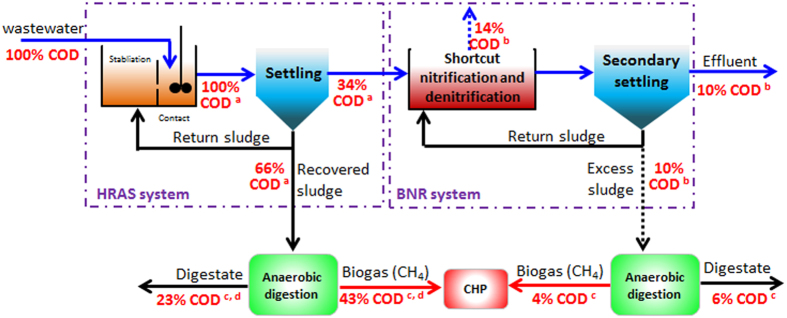
COD flow in A-B process with HRAS as A-stage and shortcut nitrification and denitrification as B-stage; (**a**) data from Meerburg *et al.*^26^; (**b**) data from Metcalf and Eddy^12^; (**c**) data from Parkin and Owen^47^; (**d**) data from Miron *et al.*^48^.

Recently, the two HRAS configurations had been investigated for the potential energy recovery from synthetic domestic wastewater^26^, of which the HRAS process with the contact and stabilization units showed a much higher COD removal as the stabilization unit is essential for maximizing COD capture^27^. This is due to the fact that the stabilization may ensure a feast-famine cycle, while exerts a selection pressure on microorganisms leading to enhanced COD adsorption and storage in the contact unit^26^,^28^. As such, the HRAS with the configuration of combined contact and stabilization should be ideal for A-stage. Figure 5 further shows the COD mass flows in HRAS-shortcut nitrification-denitrification process. According to Fig. 5, the recoverable electrical energy can be estimated as (13.9 × 47% × 0.35) = 2.29 kJ/g COD which is significantly higher than that achieved in the conventional CAS process.

#### A3-B1 or A3-B2 process

Extensive efforts have been dedicated to explore anaerobic process for handling low strength domestic wastewater^29^,^30^,^31^,^32^. Compared with aerobic process (e.g. CAS), it has been well demonstrated that anaerobic process has advantages of no aeration needed, less sludge produced and bio-methane generation which make it more economically viable and energy-efficient. Nowadays, various anaerobic processes are available for treating domestic wastewater, e.g. up-flow anaerobic sludge blanket (UASB), fixed film anaerobic bioreactor, anaerobic baffled reactor, etc. A full scale single-stage UASB system could remove 45–75% of total COD from domestic wastewater at a HRT of 5–19 h, whereas total COD removal of 65–90% was achieved in lab-scale UASB reactor^31^,^33^, whereas 88% of total COD was removed in an anaerobic fluidized bed bioreactor (AFBR)^34^. Despite several full-scale UASB systems have been applied for domestic wastewater treatment, some technical issues still need to be resolved, including relatively long start-up period, sensitivity to seasonal temperature change, slow growth due to low organic strength in domestic wastewater etc^31^. It should be a reasonable consideration that over 80% of total COD in domestic wastewater can generally be removed in anaerobic processes. That is, if anaerobic process is selected to serve as A-stage, remaining COD should not be enough for sustaining the shortcut nitrification-denitrification in B-stage, i.e. the A3-B1 configuration might not be a feasible option in practical application. On the contrary, such low COD concentration achieved is ideal for partial nitrification coupled with anammox at B-stage, thus the A3-B2 configuration appears to be more reasonable in this sense. In fact, the process configurations similar to A3-B2 has been demonstrated to be feasible for the mainstream municipal wastewater treatment, i.e. UASB coupled with moving bed biofilm reactor (MBBR) or integrated fixed film activated sludge (IFAS) for partial nitritation-anammox^35^,^36^.

**Figure 6:**
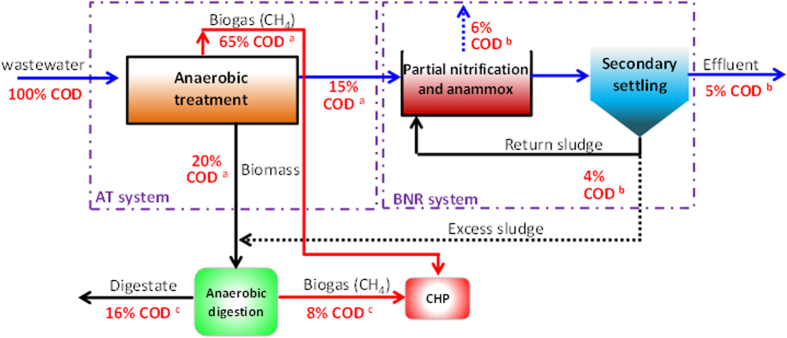
COD mass flow in A-B process with anaerobic treatment as A-stage and partial nitrification and anammox as B-stage; (**a**) data from Gopala Krishna *et al.*^37^; (**b**) data from Metcalf and Eddy^12^; (**c**) data from Parkin and Owen^47^.

The anaerobic-partial nitrification-anammox process has been identified in the above discussion as an ideal A-B process which is illustrated in Fig. 6. Highly efficient COD removal in anaerobic process provides a low COD environment for sustaining growth of anammox bacteria in B-stage. For example, 90% of total COD was removed from domestic wastewater in anaerobic baffled reactor, of which 65% of COD was converted methane^37^. According to Fig. 6, if about 65% of COD could be transformed to the COD-methane, the total recoverable electrical energy may be estimated as 13.9 × (0.65 + 0.08) × 0.35 = 3.55 kJ/g COD, which is more than the energy consumed for removing one gram COD (i.e. 3.2 kJ/g COD) in the CAS process.

## Discussion

Nowadays, extensive attention has been paid to the wastewater treatment-energy-greenhouse gas nexus in municipal wastewater treatment plant due to the serious concern on global climate change. Although various anaerobic processes as discussed above may offer perfect option as A-stage, the actual recovery efficiency of biogas from municipal wastewater through anaerobic processes is generally lower than that presented in Fig. 6 due to the dissolution of a substantial amount of methane into treated effluent. According to Yamamoto *et al.*^38^, the solubility of methane in water is about 18.6 g/m^3^ at 30 °C. This suggests that about 45% of methane produced in an anaerobic system treating municipal wastewater with a soluble COD concentration of 200 mg/L would be present in its dissolved form at 30 °C^39^. In fact, it had been reported that 50% of total methane generated eventually ended up as dissolved methane in an anaerobic MBR treating synthetic wastewater with a COD concentration of 440 mg/L at 15 °C^40^. Similar phenomenon was also observed in an expanded granular sludge blanket reactor treating domestic wastewater^41^. It has been well recognized that methane is at least 25 times more powerful than carbon dioxide in terms of the greenhouse effect. Therefore, the A-B process in Fig. 6 may be challenged by its economic viability and environmental sustainability if recovery methods for dissolved methane are not available for large-scale anaerobic treatment of low-strength domestic wastewater.

It should be pointed out that tremendous amount of sewage sludge is currently generated from WWTPs globally, e.g. about 10 million tons of dry sludge in the European Union, 8 million tons in the United States in 2010. The situation of excessive sludge in China is even more pressing due to increasing number of WWTPs countrywide, i.e. sludge production is expected to increase radically and should have reached more than 11 million tons of dry solids in 2010. The ultimate disposal of excess sludge has been and continues to be one of the most challenging and costly issues. The treatment of the excess sludge may account for 25–65% of the total plant operation cost^42^, and landfilling of dewater sludge is no longer an economically and environmentally friendly viable option. The A-B process presented in Fig. 6 indeed offers a feasible solution for ultimately solving sludge problem. In A-stage, the majority of COD in domestic wastewater is directly converted to biogas anaerobically with little sludge production. As a result, the total sludge production in the A-B process illustrated in Fig. 6 should be reduced by at least 60% compared to the conventional CAS process (Fig. 1). Given its pros and cons, a detail cost-benefit analysis of the A-B process (Fig. 6) should be further conducted in a more holistic manner.

**Figure 7:**
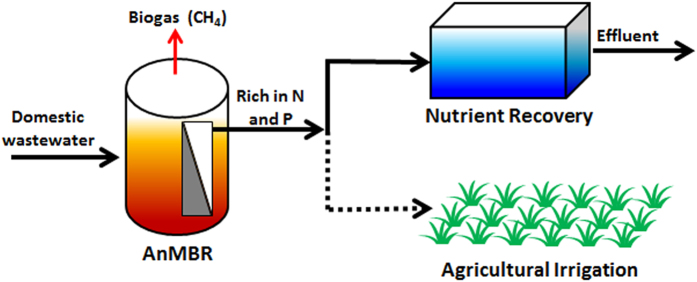
Schematic A-B process with AnMBR as A-stage and nutrient recovery as B-stage.

Recently, anaerobic membrane bioreactor (AnMBR) has attracted more and more attention for domestic wastewater treatment due to its superior performance, e.g. direct COD capture without production of much excessive sludge, extremely low energy consumption, high-efficiency biogas generation and nearly solids-free permeate^9^,^34^,^43^. Different from the A-B processes elucidated above, a new process configuration (Fig. 7) combining AnMBR and nutrient recovery may also be considered as a feasible option for concurrent energy and resources recovery from domestic wastewater. It had been shown that over 92% of COD and nearly 100% of solids removal with the minimum removal of soluble nutrients (e.g. ammonia and phosphate) could be achieved in AnMBR^32^,^38^. This suggests the possibility of nutrient recovery from the AnMBR effluent. So far, many techniques have been reported for nutrient recovery from wastewater. For example, natural zeolite as an effective adsorbent could adsorb up to 97% of ammonium, while served as seed for phosphate precipitation^44^,^45^. Moreover, zeolite synthesized from coal fly ash could concurrently remove around 60% of ammonium and 90% of phosphate present in wastewater stream^46^. It should be realized that all the methods reported for recovery of soluble nutrients from various wastewater streams are currently expensive and require strong engineering knowledge and well-trained operators in order to ensure long-term stable operation. In addition to the technology and cost issues, another obstacle in the practice of nutrient recovery from wastewater streams lies in the market acceptance, i.e. potential takers of such products in the market.

It has been actively debated if it is necessary to make all water potable. For some countries with increasing population and diverse industries (e.g. China, India, etc), the water policy should allow using different grades of water for different usages. For example, the effluent from AnMBR is nearly pathogen-free, while rich in soluble ammonia and phosphate, thus for the countries with strong agricultural industry, such AnMBR effluent may be explored for direct irrigation^9^.

## Conclusions

Although the conventional activated sludge process has been applied with great success for domestic wastewater treatment for more than 100 years, it appears that a substantial reduction in the energy consumption might not be realized through further process optimization. Indeed, as demonstrated in this study, to tackle the present energy challenge as well as tightened effluent discharge standards, we should jump out of the ivory tower of the current treatment philosophy by developing and adopting novel process configurations and emerging technologies. The solutions forward should rely on the ways to improve energy recovery, meanwhile to minimize in-plant energy consumption. In this regard, A-B processes as discussed in this study may offer the most feasible option towards energy self-sufficient municipal wastewater treatment. Making the WWTPs self-sustainable for energy demand is obviously of significance, especially when global climate change is concerned.

## Methods

**Figure 8:**
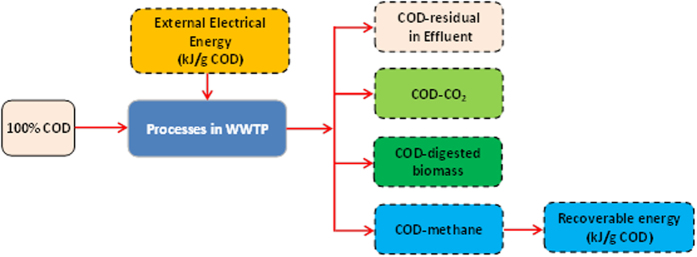
Schematic presentation of COD flux in a biological process.

In environmental engineering, COD is commonly used to measure the amount of organic maters in wastewater^10^,^11^. In this work we adopt COD in assessing the potential energy consumption and recovery in the proposed A-B processes (Figs 4, 5, 6). A typical COD concentration of 500 mg/L as commonly found in domestic wastewater was used in calculation of the mass and energy flows through the CAS and the proposed A-B processes. The COD mass flow through a specific biological process was calculated based on the reported representative COD removal efficiency in each operation unit of interest^12^,^13^,^18^,^26^,^37^,^47^,^48^. In aerobic or anaerobic process for wastewater treatment, COD is channeled into new biomass, oxidized products (e.g. carbon dioxide and methane), residual COD in the treated effluent. The biomass produced from aerobic or anaerobic degradation of COD is ultimately directed to anaerobic digester for energy recovery. In calculation of the potential energy recovery from the proposed biological processes, it is reasonably thought that energy can only be generated from methane gas produced via anaerobic degradation of directly captured COD or waste sludge as illustrated in Fig. 8. Therefore, the recoverable chemical energy from one gram COD removed can be calculated according to the theoretical chemical energy stored in one gram COD-methane, i.e. 13.9 kJ/gCOD-methane^11^. For example, for an influent COD concentration of 500 mg/L, if 43% of influent COD is ultimately converted to methane, the maximum chemical energy potentially recovered from one gram of wastewater COD can be estimated as (13.9 × 43%) = 5.98 kJ/g COD.

## Additional Information

**How to cite this article**: Wan, J. *et al.* COD capture: a feasible option towards energy self-sufficient domestic wastewater treatment. *Sci. Rep.* **6**, 25054; doi: 10.1038/srep25054 (2016).
